# Chimeric Antigen Receptor-T Cells in the Modern Era of Chronic Lymphocytic Leukemia Treatment

**DOI:** 10.3390/cancers17020268

**Published:** 2025-01-15

**Authors:** Alycia Hatashima, Mazyar Shadman, Vikram Raghunathan

**Affiliations:** 1 Department of Pharmacy, University of Washington, Seattle, WA 98195, USA; 2 Division of Hematology and Medical Oncology, University of Washington, Seattle, WA 98195, USA; 3 Fred Hutchinson Cancer Center, Seattle, WA 98109, USA

**Keywords:** CLL, CAR-T cell, chimeric antigen receptor, lisocabtagene maraleucel, immunotherapy, adoptive cellular therapy

## Abstract

Pathway inhibitors targeting Bruton tyrosine kinase (BTK) and B-cell lymphoma-2 (BCL-2) have dramatically changed the treatment landscape for both treatment-naïve and relapsed/refractory chronic lymphocytic leukemia (CLL). However, with increased utilization, a growing number of patients will experience progressive disease on both agents. This subgroup of “double refractory” patients has limited treatment options and poor prognosis. Chimeric antigen receptor (CAR)-T cells have transformed the treatment of relapsed/refractory B-cell malignancies. Although the earliest success of CAR-T cell therapy was in CLL, the clinical application of this modality has lagged until the recent approval of the first CAR-T cell product for CLL. In this review, we describe the current treatment options for upfront and subsequent therapies and the unmet need for novel agents highlighted by the burgeoning role and challenges of CAR-T cell therapy.

## Simple Summary

Common medications used to treat newly diagnosed and relapsed/refractory chronic lymphocytic leukemia (CLL) include Bruton tyrosine kinase inhibitors (BTKi) and B-cell lymphoma-2 inhibitors (BCL-2). However, once patients exhaust both options, there are a limited number of effective therapies. Chimeric antigen receptor (CAR)-T cells have become a widely used treatment modality in several B-cell malignancies and are newly being used to treat CLL. Here, we discuss the data supporting the new US Food and Drug Administration-approved CAR-T cell product for CLL, limitations of this treatment approach, and directions for future research.

## 1. Introduction

The therapeutic successes in the treatment of chronic lymphocytic leukemia (CLL) have paved a brighter future for those afflicted with the disease. As the most common adult leukemia in Western countries, over 20,000 new cases of CLL are anticipated in the United States in 2024, representing approximately one-third of all new leukemia diagnoses [1]. CLL is a malignant B-cell lymphoproliferative disease that can involve the blood, bone marrow, spleen, and lymph nodes [2]. While advances in treatment have transformed the management of this condition, CLL is considered an incurable disease without an allogeneic hematopoietic stem cell transplant (alloHSCT). The emergence of Bruton tyrosine kinase (BTK) and B-cell lymphoma 2 (BCL-2) inhibitors has largely supplanted traditional chemoimmunotherapy (CIT) both in the upfront and relapsed/refractory (R/R) settings (Table 1) [3,4]. However, challenges remain in the modern era, particularly for patients whose disease is characterized by del17p, *TP53* mutation, complex karyotype, and unmutated immunoglobulin heavy chain gene (*IGHV)* [5,6].

Historically, patients with adverse-risk CLL experienced particularly poor outcomes with short progression-free survival (PFS) and overall survival (OS) following CIT [36]. Targeted molecular therapies, with the most robust data using ibrutinib, showed efficacy in patients with del17p or *TP53* abnormalities [12,37,38,39,40]. Similarly, favorable outcomes were achieved in this difficult-to-treat population with acalabrutinib and zanubrutinib [13,14,15,22,23,41]. In addition, the first-in-class BCL-2 inhibitor, venetoclax, demonstrated response and tolerability among patients with del(17p) CLL, leading to its initial approval for this indication [42,43]. However, despite promising upfront efficacy, early relapses still occur in patients with *TP53* mutations and complex karyotype [44].

A growing percentage of CLL patients will receive both a BTK inhibitor (BTKi) and BCL-2 inhibitor, considered “double exposed”, with the expanding use of both classes of drugs: as monotherapy, in combination with anti-CD20 monoclonal antibodies, concurrently, and sequentially. This will continue to result in a burgeoning subset of individuals who progress through both agents, referred to as “double refractory” patients, with poor prognoses and limited next treatment alternatives.

At present, the optimal sequencing of antitumor therapies remains unknown for patients with “double exposed” and particularly “double refractory” CLL. In the United States, both pirtobrutinib and lisocabtagene maraleucel (liso-cel) are preferred agents for “double refractory” disease [4]. In countries where non-covalent BTKi pirtobrutinib and chimeric antigen receptor T (CAR-T) cell therapy are not yet approved for R/R CLL, third-line or later options include CIT, idelalisib plus rituximab, alloHSCT for select, fit patients, or clinical trial [3,45,46,47].

In the era of novel agents, chemoimmunotherapy would not be the treatment of choice in the multiple refractory setting, and other later-line agents such as phosphoinositide 3-kinase (PI3K) inhibitors may have limited clinical utility based on suboptimal efficacy and immune-related toxicities [16,17,48,49,50]. A real-world, retrospective series showed that among patients with “double refractory” disease, use of a PI3K inhibitor resulted in inferior objective response rate (ORR) and median progression-free survival (PFS) compared to CAR-T cell therapy [50]. Furthermore, data are lacking to suggest pirtobrutinib’s exact place in therapy. While pirtobrutinib showed promise with durable outcomes for patients with and without C481S-mutated disease, indefinite administration, and the potential for second-site mutations may limit long-term use [27,28,51,52,53,54]. The lack of clear guidance and inconsistent treatment patterns in R/R CLL highlights a substantial unmet need, opening the door to the emerging role of CAR-T cell therapy.

## 2. Covalent Bruton Tyrosine Kinase Inhibitors

### 2.1. Ibrutinib

The armamentarium of covalent BTKi includes ibrutinib, acalabrutinib, and zanubrutinib. The most robust and time-tested data are with the first-in-class, ibrutinib, and it most notably changed the treatment landscape for patients with del17p/TP53 aberrant disease [7,8,9,38,40,55,56,57,58,59]. Ibrutinib’s initial FDA approval was based on superior PFS and OS compared to chlorambucil in the front-line setting (RESONATE-2) [8]. The 24-month survival was 98% with ibrutinib versus 85% with chlorambucil, resulting in a relative risk of death 84% lower with ibrutinib (*p* = 0.001). Ibrutinib treatment resulted in an objective response rate (ORR) of 86% compared to 35% with chlorambucil; however, this was largely driven by partial responses (PRs). Only 11% of individuals treated with ibrutinib achieved a complete response (CR), which deepened to 30% and 34% at the five- and eight-year analyses [7,9]. Additional studies evaluated ibrutinib with or without a CD20-directed monoclonal antibody in treatment-naïve CLL and demonstrated similar efficacy. However, ibrutinib continued to result in low complete response rates, and tolerability proved to be challenging [55,56,57,58,59,60,61].

Ibrutinib also demonstrated efficacy in patients with R/R CLL. Ibrutinib patients experienced higher PFS rates and longer median PFS and OS compared to ofatumumab [10,11,12,62]. While response rates were in accordance with those achieved in treatment-naïve (TN) patients, they remained predominantly PRs [10,11,12].

### 2.2. Newer Generation Covalet Bruton Tyrosine Kinase Inhibitors

Both “on-target” and “off-target” effects make BTKis difficult to tolerate with the indefinite treatment durations required. Rates of ibrutinib discontinuation due to toxicities range from 12–42% in clinical trials, and real-world analyses have published rates of 63% in the frontline and up to 50% in R/R treatment settings [9,11,55,63,64,65,66,67,68,69]. This was the impetus for the development of newer BTKis with greater specificity, retained pharmacologic activity, and improved tolerability [70,71].

#### 2.2.1. Acalabrutinib

Acalabrutinib has been studied in both TN and R/R disease, mostly compared to CIT regimens [13,14,15,16,17,18]. ELEVATE-TN compared acalabrutinib with or without obinutuzumab to chlorambucil plus obinutuzumab [13,14,15]. PFS and OS rates were higher in the acalabrutinib-containing arms; the median PFS was not reached in either acalabrutinib monotherapy or with obinutuzumab (*p* < 0.0001), and the median OS was significantly longer in acalabrutinib plus obinutuzumab compared to chlorambucil plus obinutuzumab (*p* = 0.0349). Although acalabrutinib-containing regimens were effective in patients with del17p and TP53 mutated disease, adding obinutuzumab did not yield improved PFS or OS.

The ASCEND trial comparing acalabrutinib to idelalisib or bendamustine plus rituximab (BR) demonstrated a significantly longer PFS benefit using acalabrutinib, but no difference in OS for patients with R/R CLL [16,17,18]. ELEVATE-RR was the first head-to-head trial between BTKis demonstrating non-inferiority of acalabrutinib to ibrutinib in patients with del17p and/or del11q R/R CLL [19]. The median PFS and OS in the two groups were 38.4 months and not reached, respectively. CR rates, as assessed by the independent review committee, were low at 1.9% and 3% in the acalabrutinib and ibrutinib arms. However, the investigator-assessed CR rates were 9.3% and 9.8%, respectively. This trial established acalabrutinib as an effective alternative to ibrutinib with a comparatively tolerable toxicity profile [20].

#### 2.2.2. Zanubrutinib

The most recent FDA-approved covalent BTKi, zanubrutinib, was developed to optimize the pharmacokinetic/pharmacodynamic and safety profile of prior BTKis [72,73,74,75]. The SEQUOIA trial studied zanubrutinib compared to BR in older patients or patients with comorbidities without del17p with TN CLL [21,22]. Zanubrutinib showed superior median PFS and 24-month PFS rate compared to BR, but no difference in OS. Initial response rates were 94.6% and 85.3% in the zanubrutinib and BR cohorts. Seven percent of patients in the zanubrutinib arm achieved a CR, which deepened to a CR/CRi rate of 17.4% over time. A single-arm study of zanubrutinib in a cohort of patients with del17p CLL reported high PFS and OS rates, which were sustained at the 4-year follow-up. CR rates deepened from 6% at the primary analysis to 14.5% [22,23].

The pivotal phase III ALPINE trial compared zanubrutinib to ibrutinib toxicity in R/R CLL [24,25,26]. The investigator-assessed ORR was superior in the zanubrutinib cohort and numerically higher in patients with del17p and/or TP53 abnormalities. With an extended follow-up of 36.3 months, the ORR and PFS continued to favor zanubrutinib, including patients with high-risk disease [24]. Although OS was similar (HR 0.75; 95% CI: 0.54–1.05; *p* = 0.098), the 3-year OS rates were numerically higher (82.5% vs. 79.6%) in patients treated with zanubrutinib compared to ibrutinib.

## 3. Pirtobrutinib

Pirtobrutinib is the first FDA-approved noncovalent, reversible BTKi and is an attractive treatment choice given its activity against both wild type and mutated C481S in BTK [76]. The phase I/II BRUIN trial demonstrated the efficacy and safety of pirtobrutinib in heavily pre-treated R/R CLL patients who progressed or were intolerant to another BTKi [27,28]. In the primary analysis, 73.3% (95% CI: 67.3–78.7) of patients achieved a response, with four (1.6%) CRs. Among patients who were previously treated with a BTKi and BCL2 inhibitor, 70% (95% CI: 60–78.8) responded. With more than 2 years of follow-up, the ORR remained high at 72% (95% CI: 66.4–77.1%) in all patients who received a prior covalent BTKi [28]. In the subset of patients who received a first-line covalent BTKi followed by pirtobrutinib, the ORR, including partial response with lymphocytosis (PR-L), was 89.5%. The median PFS was 19.4 months (95% CI: 16.6–22.1); among patients treated with a BTKi but not a BCL2 inhibitor, the median PFS was 23 months and 15.9 months in patients who received both agents prior to pirtobrutinib. The median OS was not estimable for the overall study population or the subgroups of patients who did or did not receive a prior BCL2 inhibitor.

The results from the BRUIN trial in R/R CLL patients revealed that pirtobrutinib maintained efficacy regardless of prior BCL2 inhibitor exposure. Pirtobrutinib had an acceptable safety profile, with only slightly higher rates of grade > 3 neutropenia in patients who previously received a BCL2 inhibitor and low treatment discontinuation rates. Pirtobrutinib thus remains an important next line of therapy, particularly when deciding to sequence therapies inhibiting the BTK pathway.

## 4. Venetoclax

Following the initial 2016 FDA-approval of venetoclax for del17p CLL, the indications for venetoclax have expanded to include patients regardless of genetic alterations, with or without a CD20-targeted monoclonal antibody [3,4,77]. Venetoclax is an effective treatment for both TN and R/R CLL, has demonstrated the ability to induce durable remissions, and may be an attractive option due to its fixed-duration treatment schedules [29,30,32,33,34,35,42,43,78,79,80,81,82,83,84,85,86,87,88,89].

The safety profile of venetoclax is most commonly marked by hematologic toxicities, including neutropenia, anemia, and thrombocytopenia. However, grade 3 or higher incidences of febrile neutropenia were reported to be 3–8% [29,35,43,90,91]. Infection and gastrointestinal toxicities are also common, with rates of diarrhea at 28–45% and nausea at 18–38% [29,31,32,35,43,90]. Perhaps the most serious complication related to venetoclax initiation is tumor lysis syndrome (TLS), which is of paramount concern, especially in patients with compromised renal function. Clinical trials report rates of both clinical and laboratory-defined TLS at 1–5% [35,42,43,90]. However, grade 3 or higher TLS has been reported with combination therapies in 3–10% of patients [29,35,89]. In the real-world, rates of clinical and laboratory TLS were reported to be approximately 3% and 6%, respectively [91].

## 5. “Double Exposed” and “Double Refractory” CLL

“Double exposed” and “double refractory” patients experience poor survival outcomes and shorter time to next treatment failure/death, especially if a covalent BTKi and BCL-2i are utilized in subsequent lines of therapy compared to first and second lines of treatment [92,93,94,95,96,97].

An international retrospective study of “double exposed” patients showed poor outcomes and limited treatment options following these initial classes of drugs [95]. The ORR to prior covalent BTKi and venetoclax was 84.7% and 69.6%, respectively. Slightly over two-thirds of patients initially discontinued a covalent BTKi and venetoclax due to progression, while 25.6% and 16.8%, respectively, stopped treatment due to toxicity. Subsequent lines of therapy included a noncovalent BTKi (n = 45), covalent BTKi (n = 43), CIT (n = 23), PI3Ki (n = 24), alloHSCT (n = 17), CAR-T cells (n = 9), venetoclax retreatment (n = 6), and a range of other therapies (n = 44). ORR was highest with CAR-T cell therapy (85.7%), alloHSCT (76.5%), noncovalent BTKi (75%) and covalent (53.7%). PI3Ki and venetoclax retreatment yielded response rates of approximately 40% and CIT had the lowest response of 31.8%. The median PFS followed a different pattern: noncovalent BTKi (not reached), venetoclax retreatment (14 months), and alloHSCT (11 months). Therapies that resulted in a median PFS of five months or fewer included PI3Ki, CAR-T cell therapy, CIT, and covalent BTKi (if previous covalent BTKi was discontinued due to progressive disease).

Another retrospective analysis conducted at the Fred Hutchinson Cancer Center (FHCC) evaluated “double exposed” patients and found that the most common subsequent therapies were CAR-T cells, PI3Ki, and noncovalent BTKis [96]. The median PFS and OS in the “double exposed” cohort were 15.4 months (95% CI: 8–39) and not reached, respectively. Among “double refractory” patients, the median PFS and OS were 6.8 months (95% CI: 3–9) and 21.2 months (95% CI: 14–46), demonstrating worse outcomes compared to “double exposed” patients. These poor survival outcomes highlight a growing challenge in the management of patients with pretreated CLL.

Lew and colleagues published a small series of patients (n = 17) with double refractory CLL [98]. Fifteen patients had a del17p and/or *TP53* mutation, and all evaluable patients had a complex karyotype. In patients treated with first line venetoclax followed by second line BTKi, the ORR was 84%, including 17% CR. In contrast, patients who received first-line BTKi and subsequent second-line venetoclax achieved an ORR of 80% but no CRs. The median time to progression was similar between the second-line BTKi and venetoclax cohorts: 25 months (range: 1–55 months) and 24 months (range: 4–42 months), respectively. Additionally, while the OS was not significantly different regardless of receiving a BTKi or venetoclax in the second-line setting, the soberingly short OS of 2.9 months and 5.3 months (*p* = 0.756) demonstrates an unmet need for these sequentially resistant individuals. The lack of an established standard of care for double refractory patients opens the door to new treatment modalities, including CAR-T cell therapy.

## 6. Chimeric Antigen Receptor T Cell Therapy

CAR-T cells have altered the treatment paradigm for R/R B-cell acute lymphoblastic leukemia (ALL) and non-Hodgkin lymphomas (NHLs) [99,100,101,102,103,104,105,106,107,108,109,110,111,112,113]. These artificially engineered T cells exploit antigen-recognizing single-chain variable fragments (scFvs), and CD19 has been the most attractive and successful target antigen due to its widespread expression in B-cell malignancies (Table 2) [114]. CAR-T cell constructs have evolved to enhance CAR-T cell persistence in vivo, and all currently FDA-approved products follow second-generation designs, which include a CD3ζ intracellular activation domain coupled with a CD28 or 4-1BB costimulatory domain [115,116].

### 6.1. CD19-Targeted CAR-T Cell Therapies

#### 6.1.1. Autologous CAR-T Cell

One of the earliest successes of CD19-directed CAR-T cells was in CLL, including patients with *TP53* deficient disease [127,128,129]. To demonstrate proof-of-concept, a pilot study was conducted using anti-CD19 CAR-T cell product CTL019, later named tisagenlecleucel (tisa-cel) in 14 patients with chemotherapy refractory or resistant disease [130]. Six patients had a del17p or *TP53* deletion, and all patients received lymphodepleting chemotherapy prior to CTL019. At a median follow-up of 19 months, eight patients (57%) responded, and four (29%) had a CR. Four patients (29%) achieved a PR. Among the patients with a CR, none relapsed and had a median duration of response (DOR) of 40 months (range: 21–53), compared to 7 months (range: 5–13) in the patients with a PR. However, six patients did not respond, and all progressed within the first nine months after administration of CTL019. The estimated PFS and OS were 7 months and 29 months with 18-month PFS and OS rates of 28.6% and 71%, respectively. Further, four patients achieved undetectable measurable residual disease (uMRD) within the first three months post-CTL019, of whom three patients were alive without disease recurrence up to 53 months after CAR-T cell infusion. Mild to severe cytokine release syndrome (CRS) was reported in nine patients with four grade 4 CRS symptoms requiring admission to the intensive care unit and cytokine-directed therapy. Five patients experienced concurrent neurologic events and one case of grade 4 confusion, which subsequently resolved.

Initial encouraging data fueled efforts to replicate the same therapeutic benefits. Investigators attempted to improve results evaluating different CAR constructs, costimulatory molecules, optimal cell dose, and ratio of CD4^+^:CD8^+^-infused T cells (Table 3) [117,118,119,122,123,131,132,133,134,135,136]. Studies also found that outcomes following infusion of CAR-T cells without prior lymphodepleting chemotherapy were largely disappointing, underscoring the importance of T cell depletion to target residual leukemia and regulatory cells [119,137,138].

Piggybacking on the early success of tisa-cel, a phase II dose optimization study in R/R CLL was conducted [117]. Thirty-eight heavily pre-treated patients were randomized to receive tisa-cel 5 × 10^7^ (low dose) or 5 × 10^8^ (high dose) CAR-T cells, of whom 32 were evaluable for response (low dose; n = 13 and high dose; n = 19). Twenty-eight percent of the evaluable patients had a del17p or *TP53* abnormality or received prior BTKi, primarily ibrutinib. Only one patient had a history of venetoclax therapy. Patients who received high-dose 5 × 10^8^ CAR-T cells experienced higher ORR and CR rates of 53% and 36% compared to 31% and 15% seen with low-dose tisa-cel. The median PFS and OS were not statistically different between the two dose cohorts. Of note, the 36-month PFS and OS rates were significantly higher in patients who achieved a CR regardless of CAR-T cell dose. Among the entire cohort, 48% of patients experienced grade 1–2 CRS and 11% grade 3–4 CRS by ASTCT criteria; three patients experienced grade 3 or higher neurologic events. However, the toxicity profiles remained similar regardless of the CAR-T cell dose.

While Frey and colleagues did not report duration of response, prolonged remissions have been achieved in other studies [130]. Long-term follow-up from the National Institutes of Health reported outcomes from eight CLL patients who received anti-CD19 CAR-T cell FMC63-28Z, later known as axicabtagene ciloleucel (axi-cel) [118]. The median event-free survival was 40.5 months, and 50% of patients achieved a DOR of longer than 3 years. The longest durable remission was 99 months, a very encouraging result in a historically challenging population.

An additional CD19-targeted CAR-T cell product (JCAR014) was developed at FHCC and formulated in a 1:1 ratio of CD4^+^:CD8^+^ CAR-T cells [119,121,131,132]. Twenty-four patients with R/R CLL, including five with Richter’s transformation, received lymphodepletion followed by an infusion of JCAR014 at three escalating doses of 2 × 10^5^, 2 × 10^6^, or 2 × 10^7^ CAR-T cells/kg [119]. However, after one patient experienced grade 4 CRS and grade 3 neurotoxicity, 2 × 10^6^ CAR-T cells/kg was selected as the maximum tolerated dose. Twenty-three patients had high-risk cytogenetics, nineteen patients had progressed through ibrutinib, and six patients were refractory to venetoclax. The 4-week ORR was 71% by iwCLL criteria and 70% by iwCLL lymph node criteria. Among the five “double refractory” patients, two patients achieved partial responses by iwCLL lymph node criteria, one stable disease, one progressive disease, and one patient died from toxicities prior to disease restaging. The median PFS and OS among all CLL patients were 8.5 months and not reached, respectively. Patients who achieved a lymph node response experienced longer median PFS and OS compared to those who had stable disease or progression (PFS 1.1 months and OS 11.2 months). JCAR014 was associated with grade 1–2 CRS in three-quarters of patients and neurotoxicity in one-third of patients; one patient died from CRS and neurotoxicity. Long-term follow-up results from this study included forty-nine R/R CLL patients and ten patients with Richter’s transformation [121,131]. Including the expanded study population, the ORR by iwCLL criteria was 70% with over half of the responses being PRs. Seventeen percent of patients achieved a CR/CRi. With a median follow-up of 79.6 months, the median PFS and OS were 8.9 months (95% CI: 3.0–19.9) and 25.0 months (95% CI: 11.5–62.1), respectively [121]. The 6-year PFS and OS were 17.8% (95% CI: 9.7–32.8) and 31.2% (95% CI: 20.3–48.1). The median DOR was 18.9 months (95% CI: 9.66–55.6), and among patients who had a day 28 response, the estimated probability of a 6-year DOR was 26.4% (95% CI: 14.8–47.2). Among patients with bone marrow disease pre-CAR-T cells, 70% achieved MRD negativity by multiparameter flow cytometry and 62% by next-generation sequencing (NGS). However, there was a subset of patients (n = 6) who were MRD negative in the bone marrow but had persistent nodal disease, and these patients experienced a short median DOR of 7 months.

The most notable advancement in cellular immunotherapies for CLL was the March 2024 FDA approval of liso-cel for patients who progressed after at least two lines of therapy including a BTKi and BCL-2 inhibitor [139]. The approval was based on TRANSCEND CLL 004, a phase I/II multicenter, open-label trial [122,123,135]. One hundred thirty-seven adult patients with R/R CLL were enrolled and underwent leukapheresis, of whom one hundred seventeen received a single liso-cel infusion [122]. Patients in this study were multi-drug refractory, with a median of five prior lines of therapy, and over 80% had at least one high-risk cytogenetic marker. All patients had previously failed a BTKi and 70 patients also experienced venetoclax failure. Patients received lymphodepleting chemotherapy with fludarabine/cyclophosphamide and received liso-cel at dose level (DL) 1 totaling 50 × 10^6^ or 100 × 10^6^ CAR-T cells (DL2) formulated in a 1:1 ratio of CD4^+^:CD8^+^ T cells. In the full efficacy evaluable population (n = 96), the ORR was 48% (95% CI: 38–58) and CR/CRi was 18% (95% CI: 11–27). Among the subgroup of patients who progressed on a BTKi and failed venetoclax, the ORR and CRR were 43% (95% CI: 30–58) and 19% (95% CI: 9–32), respectively. A similar proportion of patients achieved uMRD in both the blood and bone marrow in the overall and “double refractory” subset. The median DOR among responders and overall PFS were approximately 35 (95% CI: 19.8–NR) months and 18 months (95% CI: 9.4–26.9) in the full population of efficacy-evaluable patients and 35 months (12.4–NR) and 12 months (95% CI: 12.7–26.2) in patients who failed a prior BTKi and venetoclax. Eighty-five percent of patients experienced any grade CRS but only nine percent developed grade 3 CRS, and there were no grade 4–5 events reported. Forty-five percent of patients were reported to have neurologic toxicities, of which nineteen percent of patients experienced grade 3 or 4 events. The median times to CRS and neurotoxicity were 4 days and 7 days, respectively. Prolonged treatment-related adverse effects included prolonged cytopenias (54%), hypogammaglobulinemia (15%), and grade 3 or higher infections (17%). The 24-month follow-up reflected sustained disease response and high uMRD rates without new safety signals [123]. In the primary efficacy analysis set, the ORR was 44% (95% CI: 30–58.7) and CRR/CRi was 20% (95% CI: 10–33.7). The uMRD in blood and marrow were 64% (95% CI: 49.2–77.1) and 60% (95% CI: 45.2–73.6), respectively. The overall median DOR and PFS were largely unchanged from the initial analysis at 35.3 months (12.4–NR) and 11.9 months (95% CI: 5.7–26.2). The median OS was 30.3 months (95% CI: 15–NR).

In patients with persistent or relapsed CLL after the first CAR-T cell infusion, studies have shown response following a second cycle of lymphodepletion and CAR-T cells [119,140]. Initial findings from a single center, dose escalation study using JCAR014 demonstrated that among six patients treated with a second infusion at the same (n = 1) or 10-fold higher (n = 5) dose, two patients were able to achieve a CR by PET-CT criteria and uMRD [119]. Both patients with CRs received a 10-fold higher dose of CAR-T cells. Two-thirds of patients developed CRS, and one patient experienced reversible grade 3 neurologic toxicity. These data were later expanded upon at the same institution to include an additional three patients (total of nine), who received second CAR-T cell infusions on the same protocol. The analysis showed two CRs and one PR by iwCLL criteria [140]. However, four patients had progression of disease. After an estimated median follow-up of 18 months post-second CAR-T cell infusion, the median PFS and OS were 80 days (95% CI: 37–not estimated) and 7 months (95% CI: 3–not estimated), respectively. In patients who had a response, the median DOR was 33 months. Despite all evaluated patients receiving higher doses of CAR-T cells with the second infusion, incidences of CRS and neurotoxicity remained low, and all grade 3–4 events were reversible.

#### 6.1.2. Allogeneic CAR-T Cell

Research into the use of allogeneic CD19-directed CAR-T cells is limited to small studies after allogeneic hematopoietic stem cell transplantation (alloHSCT) using donor-derived CAR-T cells [141,142]. Patients did not receive lymphodepletion prior to donor CAR-T cell infusion. Individual studies demonstrated overall response rates of 25–40%, and when combined, two-thirds of patients experienced stable or progressive disease. One patient experienced mild ocular graft-versus-host disease (GVHD) 2 years post-CAR-T cell infusion, without detectable persistence of the donor’s CAR-T cells [142].

Notably, Cruz and colleagues were the first to establish that donor-derived virus-specific T cells (VSTs) manipulated to express CD19-directed CARs could successfully expand and persist in patients with a high risk of relapse or relapsed CD19+ B-cell malignancies [141]. In a small phase I trial, post-alloHSCT patients who received VSTs with CD19-targeted CARs could achieve a response or maintain CR due to demonstrated cytotoxic and anti-viral activity. These genetically modified VSTs were reported to have a favorable safety profile as there was an absence of infusion-related reactions and GVHD due to the absence of apparent alloreactivity of VSTs.

### 6.2. CD19-Targeted CAR-T Cells + BTKi

The hypothesis that the addition of ibrutinib to CAR-T cells may improve response and clinical efficacy stems from in vitro and ex vivo observations that ibrutinib can enhance T-cell function as well as improve CAR-T cell expansion, engraftment, and survival [143,144]. Ibrutinib’s effect on T cell function may be owed to its potent inhibition of not only BTK but interleukin-2-inducible tyrosine kinase (ITK) [145]. Inhibition of ITK in immunosuppressive T helper (Th)2-type CD4^+^ T cells has been shown to enhance immune function [146]. This is supported by the lack of synergistic effect seen with zanubrutinib, which is 20x less potent compared to ibrutinib in inhibiting ITK.

To compare the impact of ibrutinib added to CAR-T cells, Gauthier and colleagues reported outcomes from a retrospective comparative analysis of patients who received JCAR014 with and without ibrutinib (Table 4) [120,147]. Ibrutinib 420 mg once daily was administered starting two weeks prior to leukapheresis until three months post CAR-T cell infusion. All patients received fludarabine/cyclophosphamide lymphodepleting chemotherapy and a dose of 2 × 10^6^ CAR-T cells/kg. All patients in the ibrutinib arm failed ibrutinib: 18 patients experienced disease progression, and one patient had stable disease. Twelve patients were receiving ibrutinib at the start of the study and seven of the patients who previously progressed on ibrutinib recommenced therapy prior to leukapheresis. At four weeks, the overall response by iwCLL criteria was 83% (ibrutinib) compared to 56% (no ibrutinib; *p* = 0.15) [120]. Similarly, a numerically higher proportion of patients achieved a response per iwCLL CT response criteria in the ibrutinib arm (71%) compared to no ibrutinib (53%; *p* = 0.46). After a median of 12 months follow-up, the one-year rates of PFS and OS were 38% (95% CI: 14–100) and 64% (95% CI: 42–98) in the ibrutinib group, and 50% (95% CI: 31–79; *p* = 0.91) and 61% (95% CI: 42–88; *p* = 0.8) in the no ibrutinib group. In patients who received concurrent ibrutinib, the one-year probabilities of PFS and OS in MRD-negative by marrow response were 55% (95% CI: 29–100) and 79% (95% CI: 56–100), respectively. In multivariate regression analysis, concurrent ibrutinib administration (OR = 14.02; 95% CI 0.52–379.61) and lower pre-treatment SUV_max_ on PET imaging (OR = 1.31 per SUV unit decrease; 95% CI 1.05–1.67) were associated with higher probability of response per iwCLL criteria [147]. Ibrutinib added to JCAR014 resulted in a numerically lower rate of all grade CRS compared to no ibrutinib and the severity was not significantly different between the groups [120]. However, grade 3 or higher CRS was statistically lower in the ibrutinib arm versus no ibrutinib (0% vs. 11%; *p* = 0.18). All grade and grade 3 or higher rates of neurotoxicity were similar between the groups. Of importance, two patients experienced serious adverse events: one patient in the ibrutinib arm developed with grade 2 CRS and died from a presumed cardiac arrhythmia; one patient in the no ibrutinib arm experienced grade 5 CRS and neurotoxicity.

In addition, a small cohort from the phase I cohort of TRANSCEND CLL 004 evaluated 19 patients who received liso-cell with ibrutinib [124,148]. Four patients received liso-cel at DL1 and fifteen patients at DL2. All patients had previously relapsed or were refractory to ibrutinib and either resumed or continued ibrutinib at enrollment. At 30 days post liso-cel infusion, 95% achieved a response with 63% in CR/CRi. uMRD in the blood and bone marrow was achieved in 89% and 79% of patients. CRS effects were predominantly mild; 32% of patients experienced neurologic toxicity, half of whom had a grade 3 or higher neurologic event. Of importance, ibrutinib-related side effects were reported: diarrhea (n = 7), hypertension (n = 4), atrial fibrillation (n = 1), and rash (n = 1).

Investigators at the University of Pennsylvania conducted a prospective, single-center phase II trial evaluating concurrent ibrutinib in patients who had not having achieved a CR after 6 or more months of ibrutinib therapy with autologous CD19-targeted humanized binding domain T cells (huCART-19) also called CTL119 [125,126]. Nineteen patients received a huCART-19 infusion and were followed for a median of 41 months. Of the 16 evaluable patients, 44% (95% CI: 23–67) of patients achieved a CR at 3 months by iwCLL criteria. Fifteen patients had uMRD at 3 or 6 months of whom 13 continued to be in CR at last follow-up. The median PFS and OS were both not reached; 4-year PFS and OS rates were 70% and 84%, respectively. Most incidences of CRS or Immune Effector Cell-Associated Neurotoxicity Syndrome (ICANS) were of low severity, except for one patient who died after suffering from grade 4 CRS and ICANS. One patient died over 2.5 years post huCART-19 infusion from possible treatment-related infection.

Similarly, Fraietta and colleagues demonstrated that antitumor benefits from CD19-targeted CAR-T cell therapy (CTL019) were achieved only after prolonged ibrutinib therapy [144]. Ibrutinib therapy continued for at least five cycles resulted in decreased expression of the inhibitory programmed cell death 1 on T cells and CD200 on B-CLL cells, which play a role in the functional CD8+ T cell response.

### 6.3. CAR-T Cell Toxicity Management

Post-CAR-T cell infusion toxicities, including CRS and neurotoxicity/ICANS, have been well described in the literature [117,118,119,120,121,122,123,124,125,126]. As such, early recognition of the signs and symptoms of CRS and ICANS is critical. Importantly, the prescribing information corresponding to each FDA-approved CAR product contains guidance for the mangement of CRS and neurologic complications. In brief, upfront interventions to treat CRS typically include a corticosteroid, most commonly dexamethasone, and anti-interleukin (IL)-6 therapy, tocilizumab [149,150,151]. Similarly, ICANS treatment consists of a corticosteroid plus anti-seizure therapy. However, when initial therapies fail, definitive next treatment modalities are unclear [152,153]. Secondary hemophagocytic lymphohistiocytosis or macrophage activation syndrome-like toxicities are also increasingly recognized as severe manifestations of hyper-stimulation of the immune system [151,153,154,155]. Published consensus guidelines and expert reviews provide comprehensive recommendations for CAR-T cell-related side effect management, which are outside the scope of this review [149,150,151,152,153,155,156,157].

### 6.4. Predictors of CAR-T Cell Response

The discrepancy in therapeutic efficacy between patients who respond to CAR-T cell therapy versus non-responders is dramatic, and efforts to characterize patient and disease-specific factors predictive of response are ongoing. Importantly, demographic and disease-related characteristics, including TP53-related mutations, do not appear to be associated with prolonged DOR or efficacy [117,118,158].

To date, data suggest that regardless of the CAR construct or enriched T cell subset, in vivo expansion of the CAR-T cells is paramount for antitumor efficacy [129,130,158]. Studies have shown that peak CAR-T cell expansion, specifically CD8^+^/EGFRt^+^ CAR-T cells, is important for disease elimination from the marrow, response and DOR [118,119,131]. MRD status and PET-CT response at day 28 post CAR-T cell infusion have also been associated with DOR [121,131].

Furthermore, intrinsic T cell fitness may be associated with efficacy, expansion, and persistence [158]. CTL019 cells in patients who achieved a CR or had highly active T cell products showed an upregulation of the IL-6/STAT3 pathway, leading to higher levels of STAT3 signaling mediators and targets. This suggests that STAT3-activated CAR-T cells may generate less-differentiated and potent T cells. IL-6/STAT3-pathway gene enrichment in CTL019 cells prior to infusion was also found to strongly correlate with the maximum degree of CAR-T cell expansion in vivo. However, there are conflicting data regarding the relationship between in vivo CAR-T cell persistence and durable remissions [118,130,131,159].

The expression of inhibitory immune checkpoints on CAR-T cells is also impactful on response outcomes. Significantly higher levels of cytotoxic T-lymphocyte-associated protein 4 (CTLA-4) and T-cell immunoglobulin and mucin domain 3 (TIM-3) or lymphocyte-activation gene 3 (LAG-3) expression were found on CAR-T cells of non-responders with CLL [160]. Furthermore, the upregulation of programmed death-1 (PD-1) and TIM-3 was associated with a reduced capacity for CAR-T cell expansion and cytotoxicity. These findings were validated by Fraietta and colleagues, who showed that pre-infusion CTL019 cells co-expressing PD-1 with both CD8^+^LAG-3^+^ and CD8^+^TIM-3^+^ led to poor responses, and patients who had a reduced proportion of these cells were able to achieve complete and durable responses [158].

Higher levels of blood-based biomarkers, such as IL-6 and IL-15, have been shown in patients who achieved a CR compared to those with no response [158]. Causative mechanisms behind these findings may include the importance of IL-6 trans signaling via the GP130/STAT3 pathway, which promotes the expansion and cytotoxicity of CAR-T cells [161]. IL-15 has been shown to play a role in the formation of CD8 memory T cells and in directing T cells to take on memory stem-like phenotypes for enhanced expansion and survival [162]. In mice, CAR-T cells cultured in vivo with IL-7/IL-15 resulted in CD8+ central memory T cells resulting in CAR-T cell proliferation and antitumor efficacy [163]. Other reports did not find an association between soluble IL-6 receptor and DOR, PFS, OS, or peak CAR-T cell expansion [121].

### 6.5. Challenges

The promise of CAR-T cell therapy in CLL has been tempered by the realization that the kinetics of T cell expansion, the composition of modified cells, and the environment into which the cells were infused differed from the host biology of patients with other malignancies, for which CAR-T cells have elicited high responses (Table 5). The prevailing theories driving lower response rates in CLL include an immunosuppressive tumor microenvironment and T cell exhaustion [164,165]. Of note, while target antigen escape has been described in approximately one-quarter of relapses in B-cell ALL and DLBCL, the comparable incidences have not been found in CLL [103,166].

**Figure 1:**
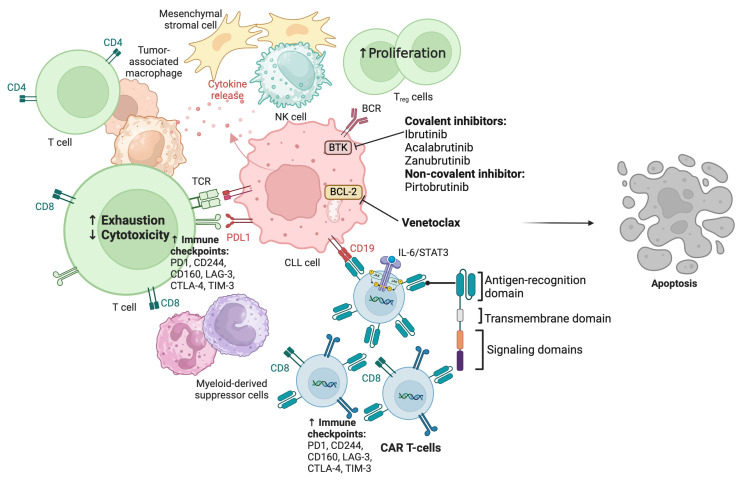
CLL tumor microenvironmental cells and cytokines leading to immune evasion by CLL cells and inhibition of CAR-T cell expansion and cytotoxicity. Approved therapeutic targets include inhibitors of BTK and BCL-2, as well as CAR-T cell therapy. CLL: chronic lymphocytic leukemia; PD-1: programmed death-1; PDL-1: programmed death ligand-1; CD: cluster of differentiation; CTLA-4: cytotoxic T-lymphocyte-associated protein 4; LAG-3: lymphocyte-activation gene 3; TIM-3: T-cell immunoglobulin and mucin domain 3; BCR: B-cell receptor; Treg: regulatory T cells; CAR-T cell: chimeric antigen receptor-T cell; IL-6: interleukin 6; STAT3: signal transducer and activator of transcription 3. Created in BioRender, Hatashima, A. (2025) https://BioRender.com/j86v246 (accessed on 9 January 2025).

A hallmark characteristic of CLL is significant immune dysregulation, leading to increased risks of infection, secondary malignancies, and autoimmune cytopenias [89,90,91,92]. CLL-induced immune dysregulation is not only limited to B cells, but also myeloid-derived immune and suppressor cells, such as tumor-associated macrophages, complement, T cells, cancer-associated fibroblasts, cytokines, and mesenchymal stromal cells, which work in concert to sustain cell-to-cell communication and support leukemic growth, as well as evade immune surveillance (Figure 1) [93,94,95,96]. In addition, monocytic myeloid-derived suppressor cells have been shown to negatively impact T cell growth and function and promote regulatory T cells [167]. Thus, the uninviting and well-curated tumor microenvironment inhibits CAR-T cell expansion and can induce a state of exhaustion and apoptosis [165].

Additionally, chronic exposure of T cells to leukemic cells may lead to defects or the absence of effector functions. CLL patients exhibit high levels of exhaustion markers such as PD-1, CD244, CD160, CTLA-4, TIM-3, and LAG-3, resulting in diminished expansion and antitumor killing as well as response to CAR-T cells [158,168].

### 6.6. Patient Reported Outcomes

Despite the enthusiasm to treat patients with novel CAR-T cell therapies, patient goals and quality of life (QOL) should be recognized as relevant outcomes, although they are at times overlooked. Although not specific to CLL, efforts have been made to characterize patient-reported outcomes (PROs) with CAR-T cell therapy [169,170,171].

Investigators at Massachusetts General Hospital studied patient-reported QOL, psychological distress, and physical symptoms at baseline and up to six months post-CAR-T cell therapy [169]. Consistent across all three scoring systems, patients experienced worsened QOL, depression, and moderate or severe physical symptoms at one week during hospitalization, which later recovered to baseline or better at six months following CAR-T cell infusion.

Another study evaluated the long-term repercussions one to five years after CAR-T cell therapy using Patient-Reported Outcomes Measurement Information System (PROMIS) measures [170]. Patients who received CAR-T cells did not experience clinically meaningful differences in PROMIS domains of neuropsychiatric symptoms of anxiety or depression, pain, fatigue, sleep disturbance, social functioning, and global mental/physical health relative to the general United States population. However, 47.5% of patients reported at least one cognitive difficulty and/or clinically meaningful depression and/or anxiety. Approximately 18% of patients scored at least one standard deviation worse than the general population mean in global mental health. In multivariate analysis, pre-CAR-T cell depression and acute grade 2–4 neurotoxicity were associated with post-CAR-T cell cognitive difficulties. Furthermore, symptoms of fatigue, anxiety, pain interference, sleep disturbance, and physical and social function were associated with increased cognitive difficulties. Increased cognitive difficulties, in turn, led to poorer global physical and mental health. The results also showed that younger age was significantly associated with elevated anxiety and depression and overall poor mental health compared to older patients, emphasizing the importance of providing mental health resources to this cohort of patients.

Given that HSCT remains a viable option for select CLL patients, it is important to consider the physical and psychiatric burden of HSCT relative to CAR-T cell therapy. When compared to autologous or allogeneic HSCT, short-term QOL, physical, and functional well-being were higher, and the impact was less severe and prolonged in patients undergoing CAR-T cell therapy [171]. Any grade and grade 3 adverse events were significantly worse on almost all PRO factors using the PRO-CTCAE Grading in patients undergoing alloHSCT. Adverse events were similar between autologous HSCT and CAR-T cell patients, except for any grade neuropathies, any grade and grade 3 diarrhea, and grade 3 pain and fatigue, which were worse in autologous HSCT patients. Furthermore, while patients who received CAR-T cells did not report a significant decline in cognitive function, patients who received autologous and alloHSCT experienced cognitive declines at two weeks and at both two weeks and one month post-transplant, respectively.

As CLL is a chronic, incurable disease, patients with R/R CLL will endure multiple lines of therapy, and studies have found that patients suffer from diminished social, emotional, and physical QOL measures throughout the treatment journey [172]. This is in addition to the protracted financial toxicities of oral targeted therapies [173,174]. The psychological and physical morbidities may, at least temporarily, continue following CAR-T cell therapy and should be considered due to the inherent risks of CAR-T cell therapy and potential need for future therapies.

## 7. CAR-NK Cell Therapy

A growing body of research into harnessing NK (natural killer) cells highlights another potential immunotherapy-based approach for the treatment of CLL [175,176,177,178,179]. NK cells are derived from lymphoid progenitor cells and participate in innate immune functions. However, a population of long-lasting memory-like NK cells can be induced with certain stimuli [180]. NK cells are able to identify target cells using a variety of receptors, most notably killer-cell immunoglobulin-like receptors (KIRs) [181]. NK cell activation results in target cell lysis via two mechanisms: the release of lytic granules and through the binding of pro-death signaling ligands to target cell receptors, activating a cascade of signaling that leads to apoptosis.

Liu and colleagues investigated the use of cord blood-derived CD19-directed CAR-NK cells in vitro and in xenograft models and found that the CAR-NK cells achieved successful expansion and antitumor effects [176]. Of note, when compared to patient-derived CAR-NK cells, the cord blood-derived CAR-NK cells achieved enhanced cytotoxic activity. This is consistent with previous findings showing that cord blood-derived NK cells have a higher proliferation capacity than peripheral blood equivalents when stimulated by exogenous cytokines [182].

Initial success using anti-CD19 or CD20 CAR-NK cells in vitro led to a phase I/II trial of eleven patients with R/R CLL and/or Richter’s transformation (n = 5) or NHL (n = 6) [177,178,179]. In the single-center, dose-escalation trial, patients were infused with anti-CD19/IL-15 CAR-NK cells derived from cord blood at doses ranging from 1 × 10^5^ cells/kg to 1 × 10^7^ cells/kg [179]. Seventy-three percent of patients responded, including 88% of responders achieving a CR. Among the patients with the diagnosis of CLL, 60% of patients achieved a CR, and 20% had remission of the Richter’s transformation but ongoing disease. The CAR-NK cells were well tolerated: there were no reports of CRS, neurotoxicity, or GVHD. The most common grade 3–4 adverse events were hematologic toxicities. The completed trial included an additional 26 patients in the expansion phase; eleven patients received 1 × 10^7^ anti-CD19/IL-15 CAR-NK cells, and the remaining patients received a flat dose of 8 × 10^8^ CAR-NK cells [183]. Including the entire phase I and II cohorts, 48.6% (95% CI: 31.9–65.6) of patients achieved a response at day +30 and +100 following CAR-NK cell infusion. Additionally, 27% (95% CI: 13.8–44.1) and 29.7% (95% CI: 15.9–47) of patients had a CR at day +30 and +100, respectively. The authors also found that patients who achieved a response at day +30 had a significantly higher 1-year OS (94.4% vs. 58.3%; *p* = 0.01) and PFS (50% vs. 25%; *p* = 0.016) compared to patients without a response. Among patients with CLL without Richter’s transformation, the day +30 overall response and 1-year CR rates were 67% and 50%, respectively.

## 8. Future Directions

The intrinsic T cell dysfunction in CLL and complex interworking of cytokine signaling within the tumor microenvironment are challenges that have been difficult to overcome with current second-generation CAR-T cell products. One strategy to theoretically improve response rates and duration is through CAR construct modifications. Single-center studies are ongoing using third-generation autologous CD19-directed CAR-T cells with two co-stimulatory domains, CD28 and 4-1BB, and fourth-generation “armored” CAR-T cells [184,185,186,187]. While conclusions are limited based on small study size, initial objective response rates were reported to be 43–50% and CR rates 0–33%. Recently published data of the third-generation Heidelberg CAR T-cell trial 1 (HD-CAR-1) product in CLL patients (n = 9) reported CR as best response in 86% of patients administered CAR-T cell doses above level 2 and uMRD in 72% of patients [187]. The median DOR was 6.4 months. The 2-year PFS and OS were 30% and 69%, respectively. Median PFS was longer in patients with a CR versus those with a PR (12.1 months vs. 3.8 months; *p* = 0.024). Seventy-eight percent of patients experienced CRS, but only 11% experienced higher-grade CRS. No patients developed signs of neurotoxicity. Of the initial cohort, three patients have since died due to disease progression, but two patients remain in an MRD-negative CR.

In addition, attempts have been made to optimize CAR-T cell products using bispecific anti-CD20 and anti-CD19 CAR-T cells [188,189]. Shah and colleagues enrolled 22 adult patients with B-cell NHL or CLL in a phase 1 dose escalation/expansion study investigating the safety and efficacy of bispecific anti-CD20, anti-CD19 (LV20.19) CAR-T cells [188]. Three patients had a diagnosis of CLL, and all achieved a response at day +28, including two CR. A separate study, which included one patient with R/R CLL demonstrated a CR following tandem CD19- and CD20-directed CAR-T cell infusions, offering another avenue for potentially curative options for CLL patients [189].

CLL cells are clonally restricted to express either λ or κ light chains. As an alternative antigen target with the hopes of sparing patients from what can be profound and prolonged hypogammaglobulinemia, efforts to construct a κ immunoglobulin light chain-targeted CAR-T cell are ongoing [190].

While still in its nascent stages, CAR-NK cell therapy is a promising permutation of traditional immunotherapy strategies [179,191]. CAR-NK cells benefit from MHC-independent recognition and killing of their targets, allowing for HLA-mismatched and cord blood-derived products. CAR-NK cell therapy offers the advantage of capitalizing on the “off-the-shelf” approach, which is currently lacking with CAR-T cells. This strategy may decrease time to infusion, which is critical as some patients die from progressive disease during CAR-T cell manufacturing periods. Furthermore, CAR-NK cells have demonstrated a relatively benign safety profile with respect to CRS and ICANS and thus far have not resulted in a GVHD response [179].

## 9. Conclusions

CAR-T cell therapy has proven effective in a range of B-cell malignancies, transforming the treatment paradigm for relapsed and refractory disease [99,100,102,103,112,192]. However, the same resounding successes have not come to fruition in CLL. The results of the TRANSCEND CLL 004 are comparatively underwhelming, though long-term follow-up data are lacking [123,135]. Of greater importance is the recognition that the disease biology of CLL is significantly different from other B-cell neoplasms, with functionally handicapped T- cells and immune mediator cells, and an uninviting tumor microenvironment that compromises the efficacy of CAR-T cells. There may also be risks of CAR-T cell therapy that are specific considerations in the CLL patient population, including the potential for secondary malignancy [193,194,195,196]. While the incidence of secondary cancers following CAR-T cells has been reported to be rare, it is unknown whether CLL patients are at an increased risk, given their underlying immune dysregulation [197,198,199,200]. Cellular immunotherapy continues to be a growing field of research exploring treatment using not only autologous anti-CD19 CAR-T cells, but also newer generation CAR constructs, bispecific CAR-T cells, CAR-NK cells, and bispecific T-cell engagers.

Ongoing efforts to expand the treatment armamentarium are robust and give hope for modernized treatment algorithms. Optimal sequencing of therapies following progression and/or intolerance to BTKis and BCL-2 inhibitors therapy remains a significant challenge, with no current consensus on subsequent lines of treatment. Prospective studies are needed to clarify the optimal role for CAR-T cell products in patients with multiply relapsed or refractory CLL, taking into consideration the unique toxicities and barriers to care that this patient population may face. Additional studies are needed to identify specific patient subgroups that may benefit most from CAR-T cell therapy. Though many unanswered questions remain, CAR-T cell therapies are a promising and novel therapeutic approach in CLL and may play a significant role in the management of this condition in the future.

## Figures and Tables

**Table 1:** Currently approved BTK and BCL-2 inhibitors for treatment-naïve and relapsed/refractory CLL.

Trial	Therapeutic Target	Drug	Line of Therapy	Patient Population	Advantages	Disadvantages
RESONATE-2 [7,8,9] RESONATE [10,11,12]	BTK (Covalent, irreversible)	Ibrutinib	TN and R/R	+/− del(17p) or *TP53* mutation	▪ Most mature efficacy data and real-world experience	▪ Indefinite therapy ▪ Low CR rates ▪ Side effect intolerance ▪ C481 binding site mutations
ELEVATE-TN [13,14,15] ASCEND [16,17,18] ELEVATE-RR [19,20]		Acalabrutinib	TN (+/− obinutuzumab) and R/R		▪ Improved toxicity profile over ibrutinib ▪ Noninferior efficacy compared to ibrutinib (R/R)	▪ Indefinite therapy ▪ Low CR rates ▪ May be substituted for ibrutinib intolerance ▪ C481 binding site mutations
SEQUOIA [21,22,23] ALPINE [24,25,26]		Zanubrutinib	TN and R/R		▪ Improved toxicity profile over ibrutinib ▪ Superior efficacy over ibrutinib (R/R)	▪ Indefinite therapy ▪ Low CR rates ▪ May be substituted for ibrutinib intolerance ▪ C481 binding site mutations ▪ Shared non-C481 mutations with pirtobrutinib
BRUIN [27,28]	BTK (Noncovalent, reversible)	Pirtobrutinib	R/R	+/− del(17p) or *TP53* mutation	▪ Activity in C481S-mutated and wild-type disease	▪ Low CR rates ▪ Shared non-C481 mutations with pirtobrutinib
GAIA/CLL13 [29] CLL14 [30,31,32] MURANO [33,34,35]	BCL-2	Venetoclax	TN (+ obinutuzumab) and R/R (+ rituximab or monotherapy)	+/− del(17p) or *TP53* mutation	▪ Time-limited therapy ▪ Possible retreatment in late relapse	▪ Possible early relapse in del(17p) or *TP53*-mutated disease ▪ Risk of TLS

BTK: Bruton tyrosine kinase; BCL-2: B-cell lymphoma 2; TN: treatment-naïve; R/R: relapsed/refractory; CR: complete response; TLS: tumor lysis syndrome.

**Table 2:** Selected CD19-directed autologous CAR-T cell structures.

Trial	CAR-T Cell Product	Initial Cell Product	Vector	Binding Domain	Hinge/ Transmembrane	Co-Stimulatory Molecules
Frey et al., 2020 [117]	CTL019 (Tisa-cel)	PBMC	Lentivirus	Murine FMC63	CD8/CD8	CD3ζ/4-1BB
Cappell et al., 2020 [118]	FMC63-28Z (Axi-cel)	PBMC	Retrovirus	Murine FMC63	CD28/CD28	CD3ζ/CD28
Turtle et al., 2017 [119] Gauthier et al., 2020 [120] Liang et al., 2023 [121]	JCAR014	Defined CD4^+^:CD8^+^ central memory T cells	Lentivirus	Murine FMC63	IgG4/CD28	CD3ζ/4-1BB
Siddiqi et al., 2023 [122,123] Wierda et al., 2020 [124] (TRANSCEND 004)	JCAR017 (Liso-cel)	Defined CD4^+^:CD8^+^	Lentivirus	Murine FMC63	IgG4/IgG4	CD3ζ/4-1BB
Gill et al., 2018 [125] Gill et al., 2022 [126]	CTL119 (huCART-19)	PBMC	Lentivirus	Humanized scFv	CD8/CD8	CD3ζ/4-1BB

Tisa-cel: tisagenlecleucel; PBMC: peripheral blood mononuclear cell; axi-cel: axicabtagene ciloleucel; liso-cel: lisocabtagene ciloleucel; huCART-19: autologous anti-CD19 humanized binding domain T cells; scFv: single-chain variable fragment.

**Table 3:** Selected CD19-directed autologous CAR-T cell trials without Ibrutinib for CLL.

Trial	Number of Patients	CAR-T Cell Product	*TP53* Abnormality/ Complex Karyotype	Prior BTKi Exposure/Failure	Prior Venetoclax Exposure/Failure	ORR/CR	Median PFS/OS
Frey et al., 2020 [117]	32	CTL019 (Tisa-cel)	28%/NR	28%/NR	3%/NR	44%/28%	1 month/ 64 months
Cappell et al., 2020 [118]	7	FMC63-28Z (Axi-cel)	NR/NR	NR/NR	NR/NR	86%/57%	NR/NR
Turtle et al., 2017 [119] Liang et al., 2023 [121]	32 47	JCAR014	58%/67% NR/75%	100%/79% 96%/NR	25%/25% 39%/NR	74%/21% ~6 year: 70%/6%; 11% (CRi)	8.5 months/not reached ~6 year: 8.9 months/ 25 months
Siddiqi et al., 2023 (TRANSCEND 004) [122,123]	117	JCAR017 (Liso-cel)	46%/62%	100%/88%	80%/76%	48%/18% ~2 year: 44%/20%	17.9 months/43.2 months ~2 year: 11.9 months/30.3 months

ORR: objective response rate; CR: complete response; CRi: complete response with incomplete hematologic recovery; PFS: progression-free survival; OS: overall survival; tisa-cel: tisagenlecleucel; NR: not reported; axi-cel: axicabtagene ciloleucel; liso-cel: lisocabtagene ciloleucel.

**Table 4:** Selected CD19-directed autologous CAR-T cell with Ibrutinib for CLL.

Trial	Number of Patients	CAR-T Cell Product	*TP53* Abnormality/ Complex Karyotype	Prior BTKi Exposure/Failure	Prior Venetoclax Exposure/Failure	ORR/CR	Median PFS/OS
Gauthier et al., 2020 [120]	19	JCAR014	75%/74%	100%/100%	58%/NR	83%/NR	1-year: 38%/64%
Wierda et al., 2020 (TRANSCEND 004) [124,148]	19	JCAR017 (Liso-cel)	32%/42%	100%/100%	58%/NR	95%/63%	NR/NR
Gill et al., 2018 [125] Gill et al., 2022 [126]	19	CTL119 (huCART-19)	NR/NR 32%/37%	NR/NR 100%/NR	NR/NR NR/NR	3-month: 32%/53% 12-month: 63%/37%	NR/NR 4-year: 80%/84%

ORR: objective response rate; CR: complete response; PFS: progression-free survival; OS: overall survival; NR: not reported; liso-cel: lisocabtagene ciloleucel; huCART-19: autologous anti-CD19 humanized binding domain T cells.

**Table 5:** Limitations of CAR-T cells in CLL.

**Disease-specific factors**
▪ Immunosuppressive tumor microenvironment ▪ Intrinsic T cell fitness and exhaustion ▪ Lack of long-term follow-up efficacy and safety data
**Manufacturing considerations**
▪ CAR-T cell manufacturing delays ▪ Risk of out-of-spec CAR-T cell product
**Patient-specific factors**
▪ Accessibility of commercial and investigational CAR-T cells predominantly restricted to academic medical centers in large cities ▪ Short- and long-term physical and psychosocial quality of life implications ▪ Financial toxicity

CAR: chimeric antigen receptor.
